# Potential hazards of gas generation during underwater endoscopic submucosal dissection

**DOI:** 10.1055/a-2420-8029

**Published:** 2024-10-14

**Authors:** Tatsuma Nomura, Hiroaki Kumazawa, Yoshiaki Isono, Tomonori Saito, Ayman Qawasmi, Yoshikazu Hayashi, Katsumi Mukai

**Affiliations:** 1 Department of Gastroenterology, Suzuka General Hospital, Suzuka, Japan; 2 12838 Department of Medicine, Division of Gastroenterology, Jichi Medical University, Shimotsuke, Japan; 3 Department of Medicine, Division of Gastroenterology, Samson Assuta Ashdod University Hospital, Ashdod, Israel; 4 Department of Medicine, Division of Gastroenterology, Jichi Medical University, Shimotsuke, Japan

It has been reported that endoscopic submucosal dissection (ESD) is performed under saline immersion for safer and more efficient resection 1 2 . However, the generation of bubbles during submucosal dissection poses a significant challenge by obstructing the endoscopic view. Therefore, we developed a gas-free immersion system, an advanced method enabling ESD to be conducted without compromising the visual field 3 4 .

The bubbles generated during ESD are believed to consist of hydrogen and oxygen produced through the electrolysis of water. Therefore, if these gases accumulate in significant quantities without adequate dilution, the introduction of a high-frequency electrical current may induce a hydrogen explosion ( Fig. 1 , Video 1 ). Upon exposure to high-frequency electrical energy from the device, these accumulated gases can initiate a hydrogen explosion, often accompanied by an audible sound. Fortunately, in this instance only submucosal oozing was observed following the explosion, with spontaneous hemostasis subsequently achieved. This report presents the first documented case of ESD in which muscular layer damage occurred as a result of a gas explosion during saline-immersion dissection ( Fig. 2 ).

**Fig. 1:**
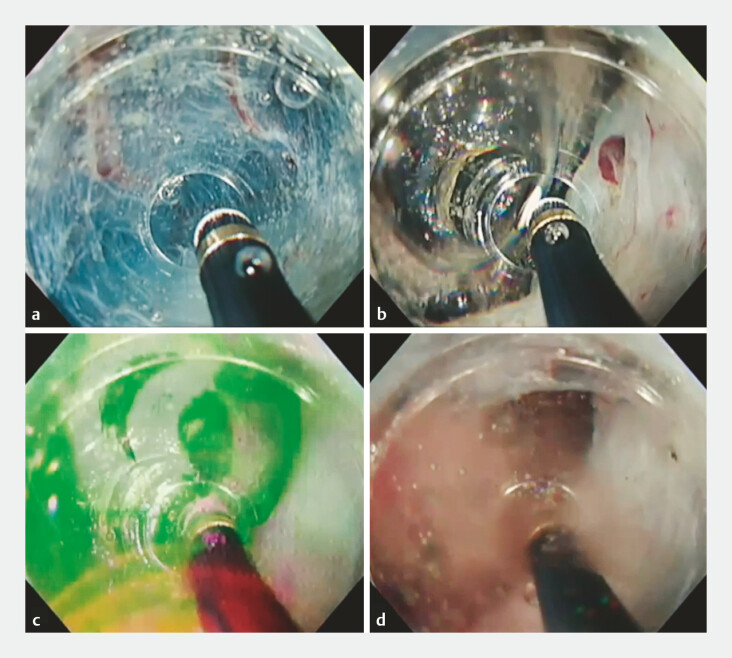
Submucosal dissection of colorectal lesion with the occurrence of a hydrogen explosion within the mucosal flap. **a** Within the saline-filled mucosal flap. **b** Bubble formation observed during submucosal layer dissection under saline (VIO 300D, swift E4-30W). **c** Accumulation of gases beneath the mucosal flap (comprising hydrogen and oxygen generated via electrolysis of water). **d** Gas produced by the explosion of the accumulated gas. The subsequent absence of gas indicates the formation of water from the reaction between hydrogen and oxygen.

**Fig. 2:**
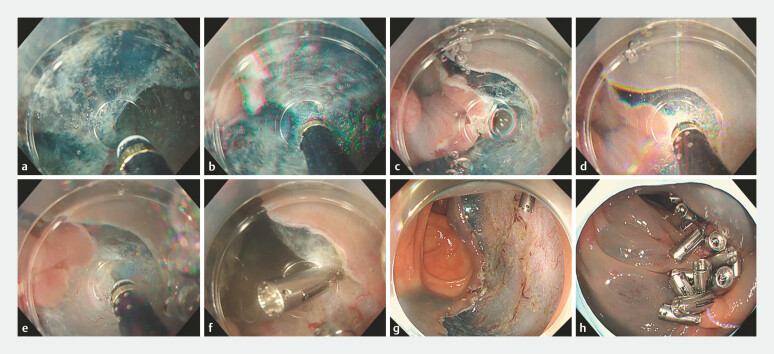
Colorectal endoscopic submucosal dissection (ESD) under immersion, demonstrating muscle layer damage induced by hydrogen explosion. **a** Saline solution filling the mucosal flap. **b** Gas generated during submucosal dissection. **c** Small gas bubbles escaping from within the mucosal flap. **d** Gas ignition during mucosal incision, leading to hydrogen explosion. **e** Minor defect in the muscle layer resulting from electrosurgical cutting with needle device. **f** Repair of damaged muscle layer with a single reopenable clip. **g** Mucosal defect observed post-colonic ESD. **h** Defect post-ESD following complete closure using reopenable-clip over the line method.

Colorectal endoscopic submucosal dissection complicated by an explosion due to gases generated during saline submucosal dissection.Video 1

The patient presented with a 50-mm tumor located in the transverse colon. Following the resection of the majority of the tumor’s central region, the peripheral margins were subsequently dissected. Unaware of the trapped gases, the endoscopist continued with the submucosal dissection, leading to a minor hydrogen explosion. While the hydrogen explosion did not directly damage the muscle layer, the force of the explosion caused oscillatory movement of the muscle layer, leading to minor trauma from the needle device. When dissection of the submucosal layer was completed, the damaged muscle area was closed using a single reopenable clip. After achieving full resection of the lesion, which resulted in a 65-mm mucosal defect, we employed the reopenable-clip over-the-line method to ensure complete defect closure 5 .

Endoscopy_UCTN_Code_CPL_1AJ_2AD_3AD

## Notes

Endoscopy E-Videos https://eref.thieme.de/e-videos
*E-Videos* is an open access online section of the journal *Endoscopy* , reporting on interesting cases and new techniques in gastroenterological endoscopy. All papers include a high-quality video and are published with a Creative Commons CC-BY license. Endoscopy E-Videos qualify for HINARI discounts and waivers and eligibility is automatically checked during the submission process. We grant 100% waivers to articles whose corresponding authors are based in Group A countries and 50% waivers to those who are based in Group B countries as classified by Research4Life (see: https://www.research4life.org/access/eligibility/ ). This section has its own submission website at https://mc.manuscriptcentral.com/e-videos .
